# An Explainable MRI-Conditioned Generative Framework for Gene-Feature Representations in Alzheimer’s Disease

**DOI:** 10.3390/ijms27219741

**Published:** 2026-10-30

**Authors:** Xiaoqing Huang, Md Hasan Imam Shihab, Ronakkumar Laxmanbhai Bhagchandani, Yuxin Dong, Yijie Wang

**Affiliations:** 1 Department of Biostatistics & Health Data Science, Indiana University School of Medicine, Indianapolis, IN 46202, USA; yuxidong@iu.edu; 2 Department of Computer Science, Indiana University Bloomington, Bloomington, IN 47408, USA; mshihab@iu.edu (M.H.I.S.); rbhagcha@iu.edu (R.L.B.); 3 Department of Mathematics, Indiana University School of Science, Indianapolis, IN 46202, USA

**Keywords:** Alzheimer’s disease, structural MRI, blood gene expression, imaging–transcriptomics, conditional variational autoencoder, molecular biomarkers

## Abstract

Magnetic resonance imaging (MRI) and peripheral-blood transcriptomics capture complementary aspects of Alzheimer’s disease (AD), but whether MRI can condition a meaningful molecular representation remains unclear. We developed and independently evaluated an explainable imaging-genetics conditional variational autoencoder (IG-CVAE) in Alzheimer’s Disease Neuroimaging Initiative (ADNI) and AddNeuroMed (ANM) cohorts using fixed 80% train-validation and 20% independent test sets with five-fold cross-validation. IG-CVAE comprises a genetic branch encoding expression into 128-dimensional gene-feature vectors (GFV128), an imaging branch encoding MRI into 128-dimensional MRI-feature vectors (MFV128), and a conditional variational autoencoder that generates GFV128 from MFV128. Paired analyses included 289 ADNI and 98 ANM participants. Test area under the receiver operating characteristic curve (ROC-AUC) values in ADNI/ANM were 0.646/0.851 for the genetic branch, 0.942/0.752 for the imaging branch, and 0.939/0.869 for generated GFV128. Generated-observed GFV128 agreement was moderate (cosine similarity, 0.590/0.589; coefficient of determination (R^2^), 0.161/0.211). Molecular importance was cohort-specific at the gene level, with no significant pathway-level convergence, whereas MRI perturbation showed cross-cohort convergence in cerebral white matter linked to distinct disease-informative molecular features. Overall, structural MRI retained disease-relevant information sufficient to generate disease-informative molecular representations, supporting IG-CVAE as a participant-paired hypothesis-generating imaging–transcriptomic framework while providing only limited evidence of participant-specific molecular correspondence without implying raw transcriptome reconstruction. The ANM test estimates should be interpreted cautiously given the small paired independent test set (*n* = 20).

## 1. Introduction

Alzheimer’s disease (AD) develops over years and is characterized by amyloid-β deposition, tau pathology, and neurodegeneration, yet individuals with similar pathology can differ greatly in cognitive decline and clinical course [1,2,3,4,5]. Thus, no single marker captures the full disease process. Structural magnetic resonance imaging (MRI) measures regional tissue loss and ventricular enlargement and supports AD detection and prediction using conventional and deep-learning methods [6,7,8,9,10]. However, MRI primarily captures structural manifestations of disease and provides limited information about the molecular processes underlying these changes. Blood-based measures provide a practical molecular view because they are minimally invasive, repeatable, and scalable [11,12]. Blood gene expression (GE) studies have reported immune, mitochondrial, metabolic, and cell-signaling alterations in AD [13,14,15,16]. Zhong et al. identified blood gene modules that classified and stratified patients and were linked to cognitive and brain-structure measures [15], while Huang et al. identified molecular subtypes and progression-related immune pathways in living participants from blood expression [16]. Blood expression, however, varies with blood-cell composition, platform, cohort characteristics, medications, and health conditions; it only partly reflects brain gene activity; and it cannot localize AD-related molecular variation within the brain [17,18,19]. Together, structural MRI and blood GE provide complementary anatomical and molecular views of AD, creating an opportunity to investigate how observable neurodegeneration relates to disease-associated molecular variation and whether these cross-modal relationships can inform molecular biomarker discovery.

Imaging-genetics studies have linked genetic variation to measurable brain features [20,21,22], and multimodal models have improved disease prediction [23,24] and gene-regulatory inference [25]. Karlsson et al. found that plasma markers best predicted tau burden and MRI’s spatial asymmetry, showing that the modalities capture complementary aspects of AD biology [23]. Studies comparing regional imaging patterns with GE atlases generally used group-level maps and postmortem tissue from different donors rather than paired, participant-level blood expression [26,27]. Thus, existing studies do not establish whether an individual’s structural brain phenotype contains information corresponding to a peripheral molecular state. Testing this relationship using participant-paired measurements is important for determining whether structural imaging can provide access to disease-relevant molecular information beyond anatomical characterization alone and for identifying molecular signatures associated with AD that warrant further investigation.

Recent generative models have begun to address cross-modal inference when one data modality is unavailable. Dolci et al. generated missing MRI and genetic features in a low-dimensional space for AD classification [28], showing that one modality could be estimated from another. However, their framework used structural and functional MRI with inherited genetic variants; it neither generated blood expression features nor tested two independent cohorts. For blood GE, directly predicting individual gene-expression values is challenging because expression is high-dimensional and sensitive to cohort- and platform-specific variation. A lower-dimensional expression-derived representation therefore provides a more stable target for testing whether MRI contains information corresponding to disease-related molecular variation without requiring exact reconstruction of individual gene measurements. Conditional variational autoencoders are suited to this setting because they learn a conditional distribution that can generate one representation from another while accounting for variability in the target representation [29]. Valid evaluation requires protecting final test data from feature selection, scaling, model training, and threshold selection to avoid leakage and optimistic estimates, particularly in small samples [30,31,32,33]. Together, these considerations motivate testing the imaging-molecular relationship in a compact latent space, where conditional generation can evaluate whether MRI contains sufficient information to generate disease-relevant molecular representation in held-out participants.

In this paper, building on the preceding evidence, we hypothesize that structural MRI contains information associated with an AD-related blood-expression-derived representation and can be used to condition its generation using participant-paired data. To evaluate this possibility, we developed the explainable imaging-genetics conditional variational autoencoder (IG-CVAE), comprising a genetic branch that learns a low-dimensional gene-feature vector (GFV128), a three-dimensional imaging branch that learns a low-dimensional MRI-feature vector (MFV128), and a conditional variational autoencoder that generates GFV128 from MFV128 in participants with paired data. The objective is to generate an MRI-conditioned representation of blood GE rather than to reconstruct individual gene-expression measurements. The complete framework was developed and evaluated independently in the Alzheimer’s Disease Neuroimaging Initiative (ADNI) and AddNeuroMed (ANM) cohorts [34,35]. We assessed agreement between observed and generated features, AD versus cognitively normal control (CN) classification, gene permutation, pathway-level organization of molecular importance, regional MRI occlusion, and projected region–gene-feature relationships. Together, these analyses test whether MRI-conditioned molecular representations contain disease-relevant information and whether the learned cross-modal relationships can be interpreted at molecular and neuroanatomical levels. Given the routine availability of structural MRI relative to blood transcriptomic profiling, such MRI-conditioned representations may also help prioritize imaging-molecular relationships for targeted molecular investigation when paired multimodal data are limited, while remaining a research tool rather than a substitute for direct molecular measurement or clinical diagnosis. Across two independent cohorts, MRI-generated GFV128 retained AD-related information despite moderate feature-level agreement and showed cohort-specific molecular importance profiles and convergent cerebral-white-matter sensitivity, supporting IG-CVAE as an approach for investigating molecular and neuroanatomical patterns relevant to AD biomarker research.

## 2. Results

### 2.1. Model-Specific Cohorts Supported Independent Unimodal and Paired Multimodal Analyses

**Figure 1:**
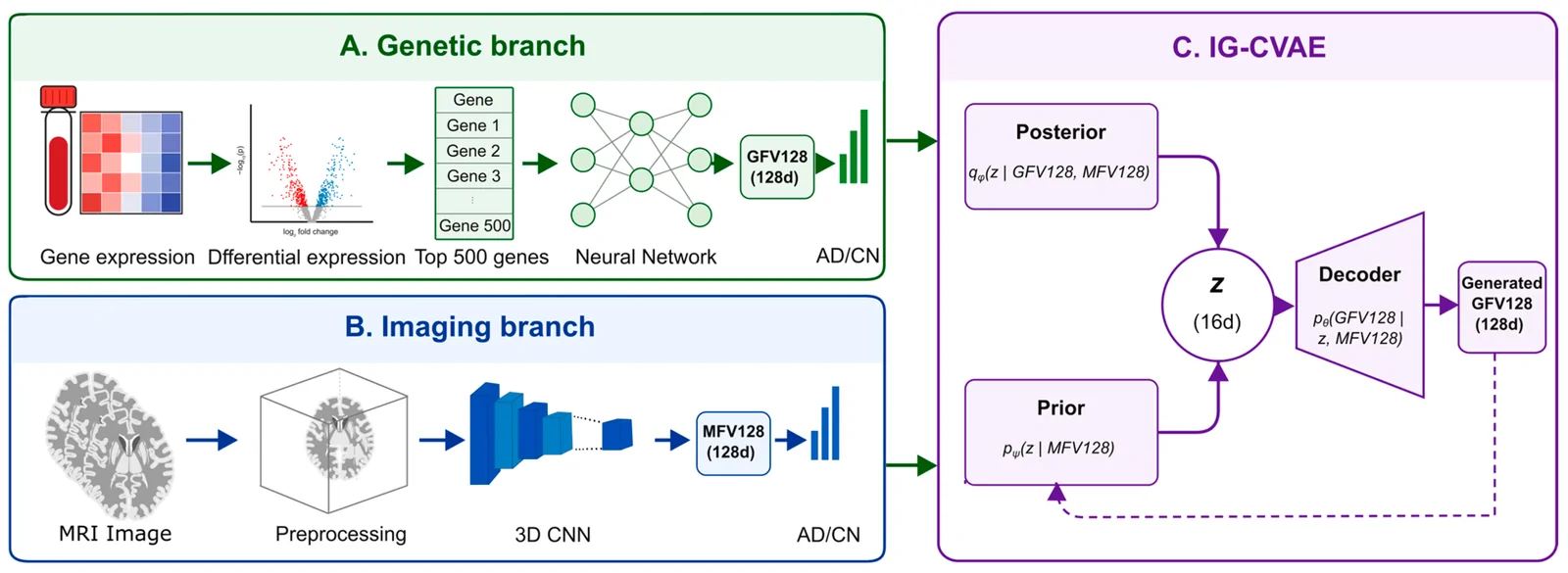
Imaging-genetics conditional variational autoencoder (IG-CVAE) framework. (**A**) The genetic branch encodes gene expression (GE) into a 128-dimensional gene-feature vector (GFV128) and predicts Alzheimer’s disease (AD) versus cognitively normal control (CN) status. (**B**) The imaging branch encodes structural magnetic resonance imaging (MRI) using a three-dimensional convolutional neural network (3D CNN) into a 128-dimensional MRI-feature vector (MFV128) and predicts AD/CN status. (**C**) IG-CVAE uses an MFV128-conditioned prior and a joint GFV128–MFV128 posterior to learn a latent representation z, from which the decoder generates GFV128 conditioned on MFV128. d, dimensionality. The dashed connector denotes the reconstruction relationship used during training. Prepared using *Inkscape 1.4.2* (*ebf0e940*, 8 May 2025).

To establish the participant sets available for each stage of the study, we first defined the modality-specific gene-expression, MRI, and paired MRI–gene-expression cohorts in ADNI and ANM. Participant characteristics and multimodal availability are summarized in Table 1, and the workflow is shown in Figure 1. Effect sizes are additionally reported in Table 1 to characterize the magnitude of AD-CN differences independently of statistical significance. Because eligibility depended on modality, train-validation/test counts in ADNI and ANM were 242/60 and 280/70 for the genetic branch, 1196/299 and 89/22 for the imaging branch, and 231/58 and 78/20 for the paired IG-CVAE analysis, respectively. In the train-validation sets, AD/CN counts were 64/178 and 145/135 for the genetic branch, 502/694 and 45/44 for the imaging branch, and 60/171 and 37/41 for IG-CVAE. Fold-specific training and validation sample sizes and AD/CN compositions for each model component and cohort are reported in Supplementary Table S1. This modality-specific design allowed the genetic and imaging branches to use all eligible participants with gene-expression or MRI data, respectively, while IG-CVAE was restricted to participants with paired measurements to learn the imaging–molecular relationship from participant-paired measurements.

### 2.2. Cohort-Specific Molecular Feature Selection Established Inputs for Multimodal Modeling

Before model training, we performed cohort-specific differential-expression ranking to define the molecular input features and finalized the gene-expression, MRI, and paired inputs for each analysis. In ADNI, 659 of 19,031 genes (3.46%) had nominal *p* ≤ 0.05, but none met Benjamini–Hochberg false discovery-rate adjusted *p* value significance (P_FDR_ ≤ 0.05); in ANM, 2072 of 4438 genes (46.69%) had nominal *p* ≤ 0.05, and 1661 (37.43%) met P_FDR_ ≤ 0.05 (Supplementary Figure S1A,B, Supplementary Tables S2 and S3). Selecting the 500 genes with the smallest diagnosis-effect *p* values within each cohort yielded genetic-branch train-validation matrices of 242 × 500 and 280 × 500 in ADNI and ANM, respectively (Supplementary Tables S4 and S5). The imaging branch received complete standardized 48 × 56 × 48-voxel inputs from 1196 ADNI and 89 ANM train-validation participants; fold-matched GFV128/MFV128 representations were available for all 231 and 78 paired train-validation participants, respectively, with no missing representations (Table 1). Because differential-expression support differed substantially between ADNI and ANM, gene selection was performed separately within each cohort rather than imposing a common panel across datasets with different transcriptomic feature spaces and signal strengths. The two top 500 panels shared 13 genes, of which 12 (92.3%) showed the same direction of differential expression across cohorts. The concordant genes were *NDUFS5*, *LSM5*, *CLC*, *COMMD3*, *DNAJC8*, *PPIH*, *TRAPPC4*, *ITGAM*, *ITGAX*, *PGS1*, *TMEM184B*, and *VCAN*, whereas *BSDC1* showed opposite directions between cohorts; cohort-specific directions and *limma* log-fold changes are provided in Supplementary Table S6. This indicates directional concordance among the shared genes despite largely cohort-specific gene prioritization. Thus, this preparation stage established complete and consistently dimensioned inputs for all three modeling components.

### 2.3. IG-CVAE Framework Captured and Preserved AD-Related Information Across Modalities

#### 2.3.1. Genetic Branch Established an AD-Informative Molecular Representation for Conditional Generation

As IG-CVAE was designed to generate GFV128 from MRI, we first evaluated whether the genetic branch produced a GFV128 representation that retained information relevant to distinguishing AD from CN in five-fold cross-validation and the independent test set (Figure 1A). Cross-validation performance was high in both cohorts: area under the receiver operating characteristic curve (ROC-AUC) and area under the precision-recall curve (PR-AUC) were 0.954 ± 0.046/0.905 ± 0.124 in ADNI and 0.888 ± 0.028/0.901 ± 0.029 in ANM (Table 2). Independent test performance was cohort-dependent, with ROC-AUC/PR-AUC of 0.646/0.602 in ADNI and 0.851/0.865 in ANM (Table 2). Thus, GFV128 encoded AD/CN-related molecular information in both cohorts, although its generalization was substantially weaker in the ADNI independent test set than in ANM. This cohort dependence is consistent with previously reported variability in blood-transcriptomic classification across ADNI and ANM [14] and motivates interpreting GFV128 as a cohort-specific molecular representation rather than a universally transferable gene-expression signature.

#### 2.3.2. Imaging Branch Established an AD-Informative Structural Representation for Conditional Generation

As IG-CVAE used MRI-derived MFV128 to condition the generation of GFV128, we evaluated whether the imaging branch produced an MFV128 representation that retained information relevant to distinguishing AD from CN (Figure 1B). In ADNI, cross-validation ROC-AUC/PR-AUC were 0.911 ± 0.012/0.877 ± 0.026 and independent test ROC-AUC/PR-AUC were 0.942/0.922 (Table 2). In ANM, cross-validation/independent test ROC-AUC/PR-AUC were 0.776 ± 0.064/0.812 ± 0.082 and 0.752/0.720, respectively (Table 2). Thus, MFV128 captured AD/CN-related structural information in both cohorts, with substantially stronger and more stable performance in ADNI. In ANM, interpretation remained limited by the small imaging sample and wide independent-test confidence intervals, consistent with the uncertainty expected in predictive neuroimaging studies with limited sample sizes [30].

#### 2.3.3. MRI-Generated GFV128 Retained AD-Related Molecular Information Despite Moderate Feature-Level Agreement

Having established that the genetic and imaging branches produced informative GFV128 and MFV128 representations, we next evaluated whether IG-CVAE could generate GFV128 from MRI that corresponded to observed GFV128 and retained information relevant to AD/CN discrimination (Figure 1C). Feature-level agreement was moderate: cross-validation cosine similarity was 0.702 ± 0.053 in ADNI and 0.650 ± 0.031 in ANM, while independent test cosine similarity/coefficient of determination (R2) was 0.590/0.161 and 0.589/0.211, respectively (Table 3). Mean squared error (MSE) and mean absolute error (MAE) are also reported in Table 3. Generated GFV128 nevertheless retained substantial AD/CN-discriminative information, with cross-validation ROC-AUCs of 0.938 ± 0.040 and 0.732 ± 0.077 and test ROC-AUCs of 0.939 and 0.869 in ADNI and ANM, respectively (Table 2). In ADNI, the cross-validation and independent test estimates were essentially identical. In ANM, the higher independent test ROC-AUC should be interpreted cautiously because the paired test set was small and the corresponding confidence interval was wide. To assess correspondence beyond shared diagnostic status, a diagnosis-matched participant-pair permutation analysis was additionally performed, in which GFV128 training targets were permuted within diagnosis groups and IG-CVAE was retrained. Models trained with correct MRI-GFV pairing showed better correspondence than models retrained after diagnosis-matched pair shuffling for MSE, cosine similarity, and R^2^ in both cohorts, although the improvement relative to shuffled pairing was modest (cross-validation R^2^: ADNI, 0.3875 vs. 0.3843, ΔR^2^ = 0.0032; ANM, 0.2983 vs. 0.2866, ΔR^2^ = 0.0117; Supplementary Table S7, Supplementary Method S1). In a sensitivity analysis removing linear effects of age, sex, and apolipoprotein E (APOE) ε4 from MFV128 and GFV128, generated–observed feature correspondence remained broadly similar, while AD/CN discrimination was attenuated but remained substantial in both cohorts (adjusted cosine similarity/R^2^/ROC-AUC: ADNI, 0.605/0.223/0.898; ANM, 0.607/0.211/0.828; Supplementary Table S8, Supplementary Method S2). As an additional cross-modal mapping benchmark, IG-CVAE was compared with a deterministic multilayer perceptron mapping from MFV128 to GFV128. IG-CVAE showed better independent-test generated–observed feature agreement across MSE, MAE, cosine similarity, and R^2^ in both cohorts, with a larger difference in ADNI and a more modest difference in ANM (R^2^: ADNI, 0.1613 vs. 0.0129; ANM, 0.2113 vs. 0.1873; Supplementary Table S9, Supplementary Method S3). Because IG-CVAE was trained and evaluated separately within each cohort, these results represent within-cohort evaluation in two independent datasets rather than formal cross-cohort model transfer. Thus, MRI-generated GFV128 preserved disease-related information represented in the blood-expression-derived feature space despite only moderate correspondence with the observed GFV128. These findings support interpreting the generated output as an MRI-conditioned molecular representation rather than a reconstruction of the participant’s measured blood transcriptome. This distinction is consistent with prior multimodal generative work showing that cross-modal latent representations can retain classification-relevant information without exactly recovering the unavailable modality [28].

### 2.4. Molecular Perturbation Revealed Cohort-Specific Gene Importance Without Pathway-Level Convergence

**Figure 2:**
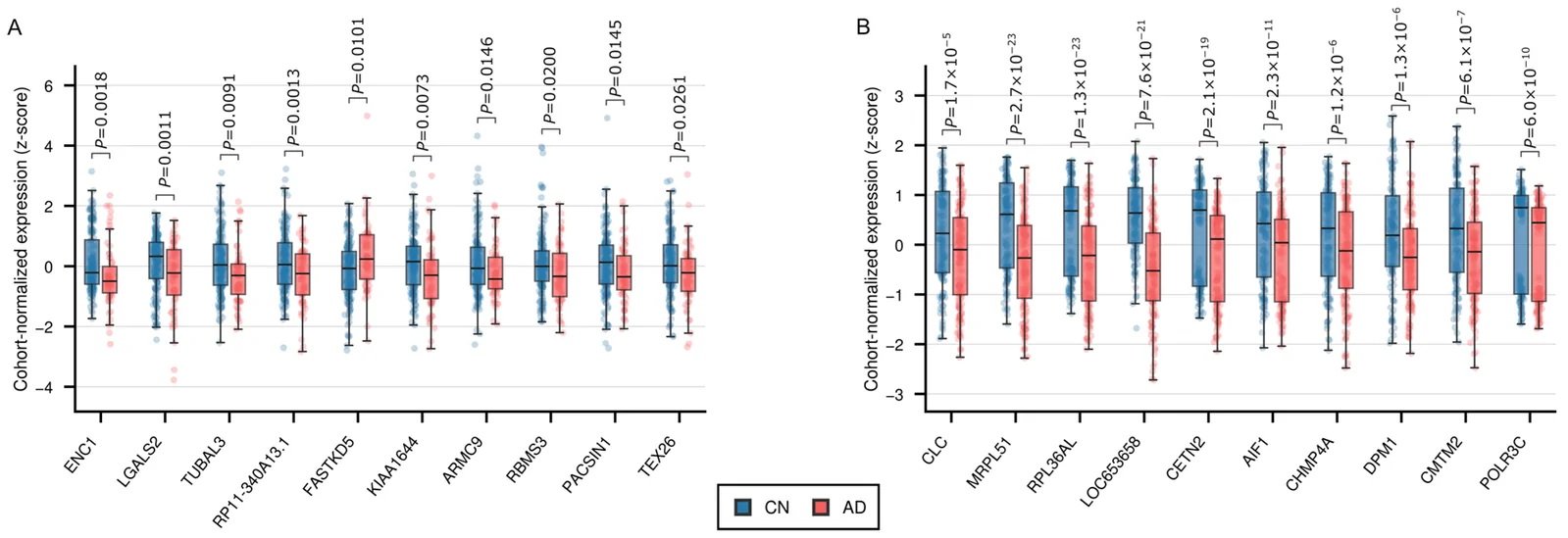
Molecular features prioritized by genetic-branch permutation importance in (**A**) the Alzheimer’s Disease Neuroimaging Initiative (ADNI) and (**B**) AddNeuroMed (ANM). Boxplots show cohort-normalized gene expression (z score) in cognitively normal controls (CN) and participants with Alzheimer’s disease (AD). Nominal *p* values were obtained from covariate-adjusted differential-expression models. Prepared using Python (*Matplotlib 3.10.3*).

To identify the molecular inputs that contributed most strongly to AD-related prediction, we examined perturbation results from the held-out validation folds in each cohort. Gene importance was cohort-specific, with no overlap among the top 20 genes: ADNI leaders were *ENC1*, *LGALS2*, and *TUBAL3* (largest ROC-AUC decrease, *ENC1*, 0.0034), whereas ANM leaders were *CLC*, *MRPL51*, and *RPL36AL* (*CLC*, 0.0115) (Figure 2A,B, Supplementary Table S10). Thus, the strongest disease-informative molecular features differed between the two independently analyzed cohorts rather than defining a common gene-level signature. To determine whether these cohort-specific gene-importance profiles converged at the pathway level, we examined pathway enrichment; no Gene Ontology Biological Process term met the P_FDR_ < 0.05 significance threshold in either ADNI or ANM (Supplementary Table S11). Thus, the cohort-specific molecular importance observed at the gene level did not resolve into a reproducible pathway-level signature. This cohort dependence is consistent with prior reports of variable blood-expression signatures and predictive performance across ADNI and ANM [14].

### 2.5. MRI Perturbation Revealed Cross-Cohort Convergence in Cerebral White Matter Associated with Cohort-Specific Molecular Features

**Figure 3:**
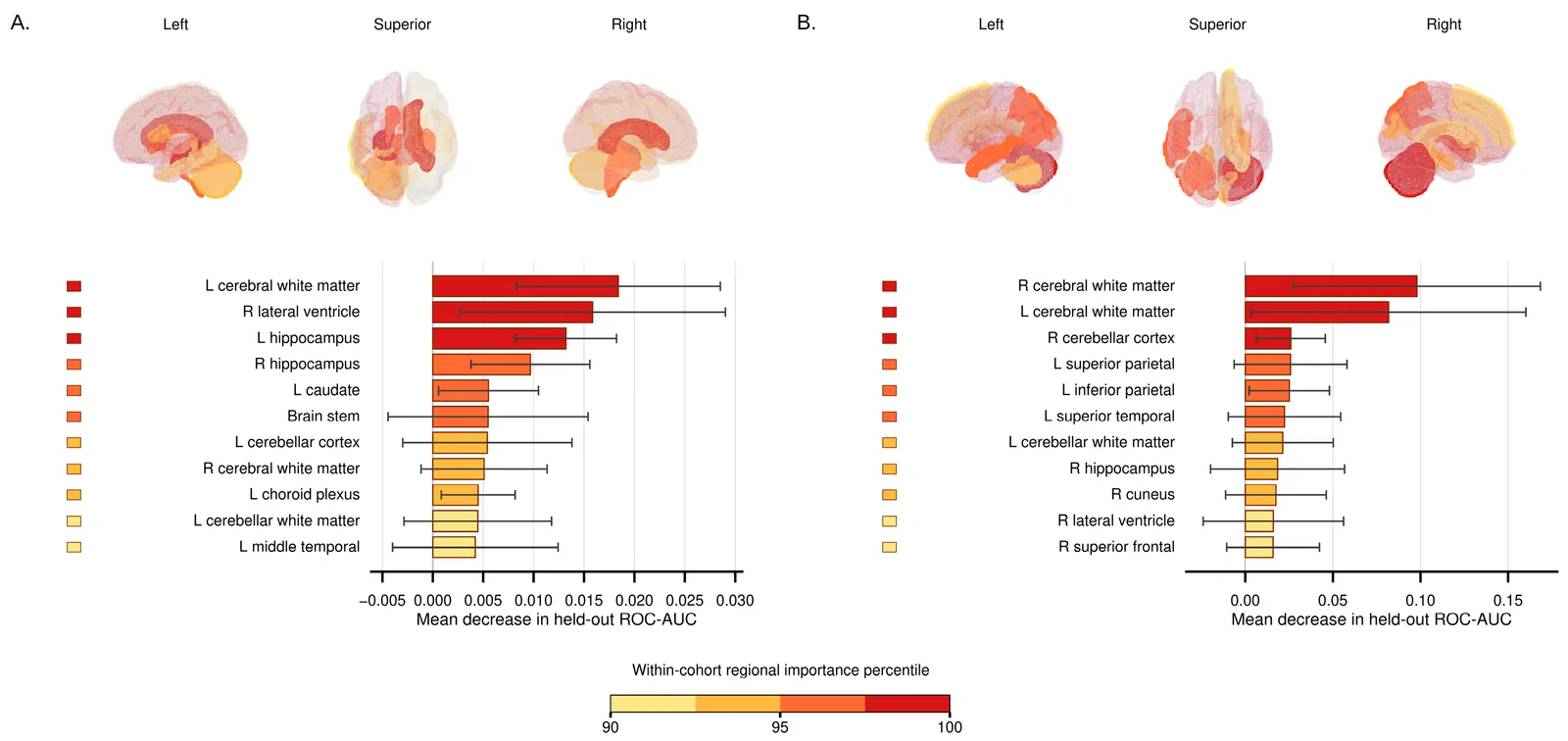
Regional imaging-branch sensitivity to zero occlusion in (**A**) the Alzheimer’s Disease Neuroimaging Initiative (ADNI) and (**B**) AddNeuroMed (ANM). Brain maps show within-cohort regional importance percentiles, and bar plots show the mean decrease in held-out receiver operating characteristic area under the curve (ROC-AUC) for the highest-ranked regions. L, left; R, right. Prepared using Python (*Matplotlib 3.10.3 and NiBabel 5.4.2*).

We next asked whether MRI perturbation identified a reproducible anatomical substrate and how that substrate related to disease-informative molecular features. At the neuroanatomical level, MRI zero-occlusion showed cross-cohort convergence in cerebral white matter. Left cerebral white matter ranked first in ADNI and second in ANM (ROC-AUC decreases, 0.0184 and 0.0818), while right cerebral white matter ranked eighth and first, respectively (0.0051 and 0.0980) (Figure 3A,B, Supplementary Table S12). Hippocampal, ventricular, and cerebellar regions also ranked highly, with cohort-specific ordering and laterality. In contrast to the gene-level and pathway-level molecular findings, these results indicate greater cross-cohort reproducibility of anatomical importance.

**Figure 4:**
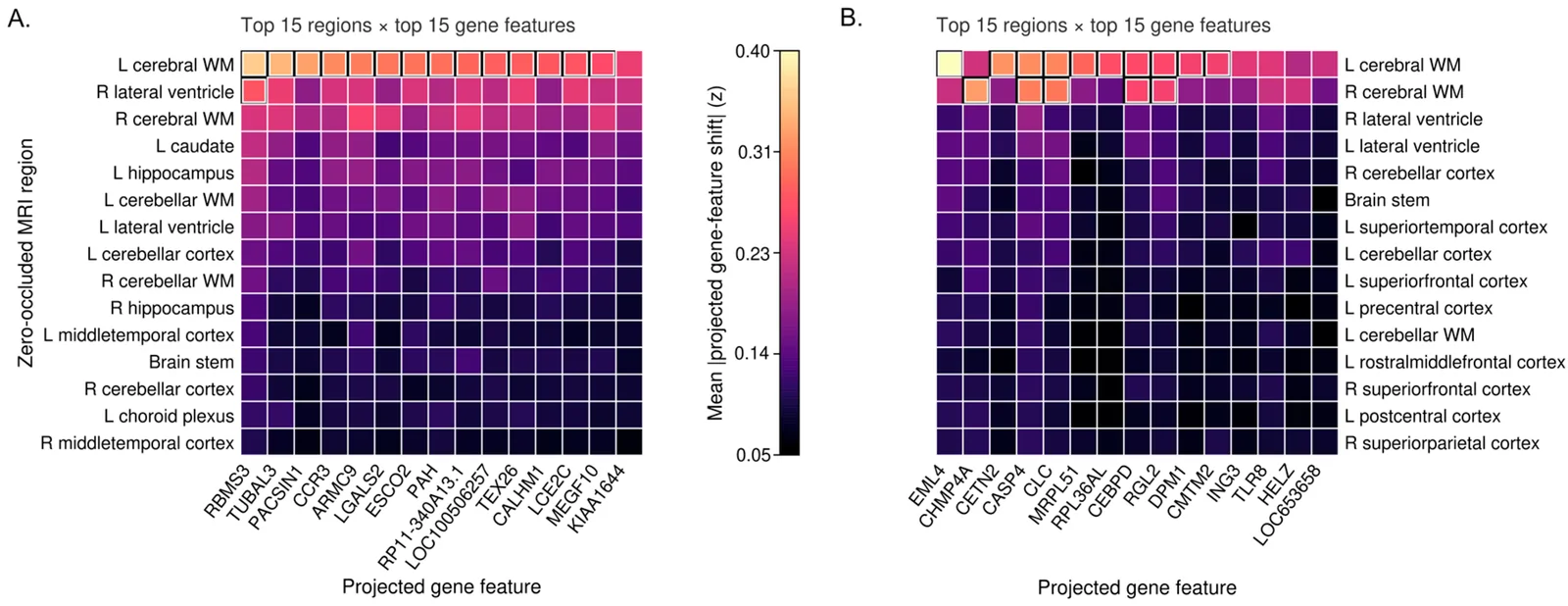
IG-CVAE region–gene-feature perturbation profiles in (**A**) the Alzheimer’s Disease Neuroimaging Initiative (ADNI) and (**B**) AddNeuroMed (ANM). Heatmaps show the mean absolute standardized shift in model-projected gene features after zero occlusion of each magnetic resonance imaging (MRI) region. Gene features were drawn from the 20 genes prioritized by genetic-branch permutation importance; rows and columns show the 15 highest-ranked MRI regions and genes, respectively. These effects represent model-derived associations within the learned MRI–GFV relationship and may partly reflect AD-related information shared across the two modalities; they do not establish region-specific gene expression or regulation. IG-CVAE, imaging-genetics conditional variational autoencoder; WM, white matter; L, left; R, right. Prepared using Python (*Matplotlib 3.10.3*).

Integrative IG-CVAE perturbation further evaluated associations between the shared white-matter signal and disease-informative molecular features. Among the top 20 genes prioritized by genetic-branch permutation importance, occlusion of cerebral white matter produced prominent shifts in model-projected gene features. The largest left-cerebral-white-matter-associated shifts involved *RBMS3* in ADNI (0.3637) and *EML4* in ANM (0.4009) (Figure 4A,B, Supplementary Table S13), with additional region–gene-feature relationships remaining cohort-specific. Together, these analyses identified a reproducible neuroanatomical pattern centered on cerebral white matter that was associated within the learned cross-modal representation with distinct disease-informative peripheral molecular features. This cross-cohort white-matter convergence is consistent with established white-matter alterations in AD [36].

## 3. Discussion

This study tested whether structural MRI contains information related to peripheral blood-expression-derived molecular variation in Alzheimer’s disease using participant-paired MRI and blood transcriptomic measurements. Rather than demonstrating exact recovery of molecular features, the findings indicate that disease-relevant information can be preserved across modalities while showing different levels of cross-cohort reproducibility. The diagnosis-matched participant-pair permutation analysis further indicated that correct MRI-GFV pairing contains some correspondence beyond shared diagnostic status, although this participant-specific component was limited relative to the overall disease-related signal. Molecular importance was largely cohort-specific, whereas cerebral white matter showed greater anatomical consistency across cohorts, suggesting that neuroanatomical signals may be more reproducible than individual peripheral molecular features in this setting. These findings position IG-CVAE as a hypothesis-generating framework for studying imaging-molecular relationships in AD rather than as a tool for reconstructing measured blood transcriptomes.

The molecular findings were strongly cohort-dependent. Prior blood-expression studies have reported immune, mitochondrial, and metabolic alterations in AD, but the specific gene signatures and predictive performance have varied across datasets [13,14,15,37,38]. Consistent with this variability, ANM showed a substantially larger proportion of genes meeting nominal and FDR significance thresholds and higher independent-test genetic-branch performance, whereas ADNI showed fewer genes meeting these statistical thresholds and a larger decline from cross-validation to independent-test performance. These differences should not be interpreted as evidence of stronger underlying biological signal in ANM because cohort composition, assay platform, leukocyte composition, sample handling, statistical power, and other sources of heterogeneity may contribute to the observed proportions [17,18,39]. Similar cohort-dependent behavior has previously been reported for blood-expression-based AD classification in ADNI and ANM [14]. The present study does not aim to improve expression-only classification; rather, it addresses a different cross-modal question by testing whether structural MRI can condition a blood-expression-derived representation using participant-paired data. Despite the limited overlap between the two top 500 gene panels, 12 of the 13 shared genes showed the same direction of differential expression across cohorts, suggesting that limited gene-level overlap can coexist with directional consistency among jointly prioritized genes. The concordant set included *NDUFS5*, *ITGAM*, and *ITGAX*; prior blood transcriptomic studies have reported mitochondrial and immune alterations in AD, including upregulation of *ITGAM* and *ITGAX* [13], with more recent work also identifying immune-associated pathways in AD blood [15]. Because gene selection was performed independently in each cohort, however, differences in prioritized molecular features may reflect both cohort heterogeneity and differences in the gene sets available to each model; consequently, limited gene-level overlap cannot be interpreted solely as biological non-replication. Overall, the present findings do not establish a reproducible cross-cohort molecular signature; rather, the prioritized molecular features should be interpreted as cohort-specific hypotheses requiring further validation.

The imaging branch was more stable in ADNI, consistent with established MRI patterns of medial-temporal atrophy, ventricular enlargement, and distributed tissue loss [6,10]. Limited ANM sample size likely contributed to greater uncertainty in both cross-validation and independent-test performance, consistent with the instability expected in predictive models evaluated in small samples [30,40]. Although ANM retained some ranking ability, its cross-validation-derived threshold classified all 22 test participants as CN, indicating that its operating threshold did not generalize reliably despite non-random ranking performance. IG-CVAE nevertheless showed a different pattern: MRI-generated GFV128 retained substantial AD/CN-discriminative information even though correspondence with the observed GFV128 was only moderate. The higher ANM independent-test ROC-AUC should not be interpreted as evidence of superior performance, given the small paired test set and corresponding wide confidence interval. The diagnosis-matched permutation analysis showed that correct participant pairing provided a small but measurable improvement in feature-level correspondence beyond diagnosis alone, whereas AD/CN discrimination was largely preserved when participant pairing was disrupted. Together, these findings indicate that generated GFV128 predominantly preserves disease-related structure shared across the diagnosis-supervised MRI and gene-expression representations, with a more limited participant-specific component; it should therefore not be interpreted as a faithful representation of an individual participant’s molecular state. This indicates that exact recovery of every latent molecular coordinate was not required to preserve the disease-related structure represented in GFV128. A similar distinction between feature reconstruction and preservation of prediction-relevant information has been observed in generative multimodal approaches that infer unavailable neuroimaging or genomic representations [28]. Accordingly, the generated GFV128 should be interpreted as an MRI-conditioned approximation of disease-relevant molecular information rather than reconstruction of an individual participant’s measured blood-expression profile.

Explainability showed stronger cross-cohort convergence at the neuroanatomical level than at the individual molecular-feature level. *ENC1*, *PACSIN1*, and *AIF1* have prior links to AD-related transcription, synaptic dysfunction, and microglial activation [41,42,43], whereas direct AD evidence remains more limited for several other highly ranked or region-linked features, including *CLC*, *MRPL51*, *RPL36AL*, *RBMS3*, *EML4*, and *CETN2*. Pathway-level analysis showed no significant enrichment in either cohort: no Gene Ontology Biological Process reached the GSEA false-discovery-rate significance threshold in either cohort. Thus, the molecular importance profiles did not converge on a reproducible pathway-level signature. This result does not exclude shared AD biology more broadly, particularly given the cohort-specific input gene panels, but indicates that the molecular signal prioritized by the models did not converge on a reproducible pathway signature.

In contrast, bilateral cerebral white matter emerged prominently in both cohorts, providing a more reproducible anatomical signal. This finding does not indicate an absence of canonical medial-temporal involvement: hippocampal regions also ranked highly, including the left and right hippocampus among the four highest-ranked regions in ADNI and the right hippocampus among the leading regions in ANM. The difference from Dolci et al. [28], who emphasized hippocampal and amygdalar gray matter, is therefore primarily one of relative anatomical prominence rather than presence versus absence of hippocampal sensitivity. Differences in model architecture, target representation, atlas definition, perturbation procedure, cohort composition, and sample size may contribute to this ordering, and the relative regional importance should therefore be regarded as model- and analysis-dependent. White-matter abnormalities in AD have been linked to myelin loss, oligodendrocyte dysfunction, axonal degeneration, and vascular injury [36,44,45]. Recent experimental evidence further suggests that myelin dysfunction may participate actively in AD pathology rather than representing only a downstream consequence: Depp et al. showed that myelin dysfunction and demyelinating injury promoted amyloid-β deposition in AD mouse models [46]. Our white-matter finding does not establish such a mechanism, but it places the cross-cohort anatomical convergence within a biologically relevant substrate increasingly implicated in AD pathophysiology. The integrative region–gene-feature analysis further showed that perturbing cerebral white matter altered model-projected features of disease-informative genes, including prominent projected shifts involving *RBMS3* in ADNI and *EML4* in ANM. These effects should be interpreted as cross-modal model associations rather than evidence that these genes are expressed in, or regulated by, cerebral white matter. Zero occlusion cannot identify the underlying biological mechanism, and confirmation will require paired imaging, molecular, and ultimately tissue-resolved validation. Thus, cerebral white matter represented the more reproducible cross-cohort substrate in our analyses, whereas the peripheral molecular features linked to that substrate remained cohort-dependent.

Limitations include comparative benchmarking being limited to a deterministic MLP cross-modal mapping baseline, with additional generative and mapping architectures remaining for future evaluation; absence of formal train-on-one-cohort/test-on-the-other transfer, meaning that evaluation in two independently modeled cohorts does not establish generalization of a trained model to an unseen external population; differences in gene-expression platforms; and small ANM imaging and paired IG-CVAE test sets [30,40]. Although the covariate-adjusted sensitivity analysis showed broadly similar MRI–GFV correspondence, age, sex, and APOE ε4 were not explicitly adjusted for in the primary imaging branch; therefore, some learned MRI features may still reflect demographic or genetic variation associated with diagnosis rather than disease-specific neuroanatomical effects alone. Thus, the study demonstrates feasibility and interpretability but not superiority over alternative mapping approaches. Cohort-specific gene panels limit direct cross-cohort molecular comparison, and blood expression is not equivalent to brain-tissue expression [19,47]. Although diagnosis-matched permutation testing supported some correspondence beyond shared AD/CN status, participant-specific molecular fidelity remained limited and requires validation in larger paired cohorts. In addition, because GFV128 is derived from transcript abundance, it does not capture DNA sequence variation or establish corresponding protein abundance or function, which may diverge from transcript levels through post-transcriptional regulation. Region–gene-feature effects therefore represent model-derived associations and cannot establish regional transcription or causal relationships. The cross-sectional design precludes temporal inference, and regional and molecular importance may depend on MRI resampling, atlas definition, zero occlusion, and ridge projection. Gene ranking was performed within the fixed train-validation partition before five-fold evaluation, making the independent test sets the strictest assessment of generalization. Future work should evaluate larger harmonized cohorts, longitudinal data, cross-cohort transfer, and biologically resolved validation of the identified imaging–molecular relationships.

## 4. Materials and Methods

### 4.1. Study Design, Cohorts, and Data Partitioning

This retrospective dual-cohort study independently analyzed AD and CN participants from ADNI (adni.loni.usc.edu) and ANM [34,48]. ADNI was launched as a public–private partnership led by Michael W. Weiner, MD, to test whether serial MRI, positron emission tomography, biomarkers, and clinical and neuropsychological assessments measure progression of mild cognitive impairment and early AD. Participant identifiers were used to match GE profiles to MRI. Participants with gene-expression data entered the genetic branch, participants with structural MRI entered the imaging branch, and participants with both modalities comprised the paired cohort used for IG-CVAE. Within each model-specific cohort, a single diagnosis-stratified 80% train-validation and 20% independent-test split was fixed before model development, with the IG-CVAE test set nested within both branch test sets. Independent test participants were held out before model development and not used for feature selection, scaling, model fitting, parameter selection, threshold selection, or early stopping. The train-validation set was assigned to five fixed stratified folds used for cross-validation and model selection. A fixed participant manifests defined eligibility, diagnosis, modality availability, and data partition for each model-specific cohort. Differential-expression ranking, scaling, model fitting, and parameter and threshold selection used only train-validation data; each independent test set was evaluated once after final model training. Independent-test 95% confidence intervals were estimated using 2000 diagnosis-stratified bootstrap replicates.

### 4.2. Gene-Expression and Structural MRI Preparation

Peripheral-blood GE data were processed separately by cohort because ADNI and ANM used different microarray platforms and workflows. Prepared gene-level, log-scale matrices were analyzed without cross-cohort batch correction. Differential-expression testing was performed once before five-fold cross-validation on 19,031 ADNI and 4438 ANM genes using only the fixed train-validation set and *limma* empirical-Bayes moderation [49], adjusting for age, sex, APOE-ε4, and, in ANM, microarray batch. Participants missing required covariates were excluded. P_FDR_ was used to characterize statistical support [50], and the 500 genes with the smallest raw diagnosis-effect *p*-values were selected separately within each cohort to define the genetic-branch input. The resulting cohort-specific 500-gene panel was fixed after selection from the train-validation data and applied unchanged to the corresponding independent test participants. T1-weighted MRI was organized in Brain Imaging Data Structure (BIDS)-compatible format and processed identically across cohorts using *fMRIPrep 25.2.3* in anatomy-only mode [51]. Preprocessing included reference image construction, intensity nonuniformity correction, skull stripping and brain mask generation, tissue segmentation, and nonlinear spatial normalization to the *MNI152NLin2009cAsym* template at 2 mm isotropic resolution. Normalized images were brain-masked, resampled to 48 × 56 × 48 voxels, and standardized within each volume. Spatial augmentation was restricted to training batches and omitted for validation and test images. Final gene-expression and MRI inputs were checked against the fixed split manifests for participant identity, diagnosis, feature order, finite values, and expected dimensions.

### 4.3. IG-CVAE Framework

#### 4.3.1. Genetic Branch

The genetic branch receives the 500-gene panel selected in Section 4.2. Within each of the five folds, a scikit-learn *StandardScaler* was fitted using only the training data and applied unchanged to the validation data. The network maps 500 standardized expression values through a 256-unit hidden layer to GFV128, followed by a 64-unit classifier layer and an AD/CN logit. Dense layers use layer normalization, Gaussian error linear unit (GELU) activation, and dropout (0.20, 0.15, and 0.10 after the 256-, GFV128-, and 64-unit layers, respectively). Training used class-weighted binary cross-entropy and *AdamW* (learning rate, 5 × 10^−4^; weight decay, 10^−4^; batch size, 32). Models were trained for up to 300 epochs with early stopping after 35 epochs without improvement in validation ROC-AUC. Fold-specific models produced held-out validation predictions and GFV128 representations; performance was summarized across folds, and pooled held-out validation predictions were used to define the AD/CN threshold. After cross-validation, final model fitting used all train-validation data for the median best epoch identified during cross-validation; the resulting model was then applied once to the test set and frozen to generate GFV128.

#### 4.3.2. Imaging Branch

The imaging branch uses a lightweight three-dimensional convolutional neural network (3D CNN) encoder. Each standardized T1-weighted MRI volume is clipped to −5 to 5 and input as a 1 × 48 × 56 × 48 tensor. Age, sex, and APOE ε4 status were not included as model inputs or regressed from the MRI-derived features; the imaging branch was intentionally defined as MRI-only to assess information contained in structural MRI itself rather than supplying demographic or genetic variables to the model. The encoder has four stages with 16, 32, 64, and 64 channels. Each stage contains two 3 × 3 × 3 convolutions with group normalization and GELU, followed by 2 × 2 × 2 max pooling. Global-average pooling reduces the final 64 feature maps to 64 features; a linear layer with layer normalization and GELU produces MFV128, and a 64-unit classifier outputs the AD/CN logit. The network uses no pretrained weights or residual connections and processes whole volumes rather than atlas-derived regions. The imaging branch used the same folds and was optimized using class-weighted binary cross-entropy with *AdamW* (learning rate, 10^−4^; weight decay, 10^−4^; batch size, 8). Training was performed up to 60 epochs with early stopping after 15 epochs without improvement in validation ROC-AUC. Fold-specific models produced held-out validation predictions and MFV128 representations. After cross-validation, a final model was trained on all train-validation data for the mean optimal epoch count identified across the five folds, then applied once to the test set and frozen to generate MFV128. The architecture was designed as a lightweight whole-volume 3D convolutional network, informed by prior 3D CNN approaches for structural MRI classification [52,53] rather than reproducing a single published architecture.

#### 4.3.3. IG-CVAE Conditional Generation

IG-CVAE comprises the genetic and imaging branches plus a conditional variational autoencoder. The fold-matched frozen branches transform GE and MRI inputs into paired GFV128 and MFV128 representations, enabling the conditional module to generate GFV128 from MFV128 while ensuring that held-out validation participants do not contribute to upstream branch training. MFV128 and GFV128 were standardized using separate scikit-learn *StandardScaler* objects fitted only on the corresponding training data and applied unchanged to held-out data. The module comprises a joint posterior *q_ϕ_* (*z*|GFV128, MFV128), an MRI-conditioned prior *p_ψ_*(*z*|MFV128), a 16-dimensional latent variable *z*, and a decoder *p_θ_*(GFV128′|*z*, MFV128). The prior maps MFV128 through two 128-unit layers, whereas the posterior maps concatenated GFV128 and MFV128 through 256- and 128-unit layers; both output 16-dimensional means and log-variances. During training, a sample from the posterior is concatenated with MFV128 and decoded through 256- and 128-unit layers to generate GFV128′. Hidden layers use layer normalization and GELU. The loss (Equation (1)) combines mean squared error (MSE) between observed and generated GFV128 with the Kullback–Leibler (KL) divergence between the posterior and conditional prior.

ℒ = MSE(GFV128, GFV128^′^) + *βD*_KL_[*q_ϕ_*(*z* |GFV128, MFV128)‖*p_ψ_*(*z*| MFV128)] (1)

Training used AdamW (learning rate, 3 × 10^−4^; weight decay, 10^−4^; batch size, 32) for up to 300 epochs with early stopping after 40 epochs without validation improvement. The KL coefficient was increased linearly from 0 to 0.01 over 40 epochs. The learning rate was reduced when validation MSE plateaued, and gradients were clipped at norm 5.0. For validation and test participants, observed GFV128 was withheld from the conditional generator; deterministic generation used MFV128 and the prior mean, *z* = *μ_p_*. Generated-observed GFV128 agreement was quantified by MSE, mean absolute error (MAE), cosine similarity, and *R*^2^, with *R*^2^ calculated as a single pooled coefficient of determination after flattening the participant-by-feature (N × 128) observed and generated GFV matrices. A separate balanced logistic-regression classifier was fitted to observed GFV128 from paired training participants and applied to MRI-generated GFV128 from the corresponding held-out participants to assess retained AD/CN information. The classifier was therefore trained on observed molecular representations and evaluated on generated representations rather than retrained on generated GFV128. After cross-validation, a final IG-CVAE was trained on all paired train-validation participants for the mean optimal epoch count and applied once to the test set. Generated outputs were interpreted as an MRI-conditioned gene-feature representation, not reconstructed gene expression.

### 4.4. Gene Permutation Importance and Pathway-Level Analysis

Explainability analyses used frozen fold-specific models and were performed exclusively on held-out validation folds, with results averaged across five folds; independent test participants were excluded from all explainability analyses. Gene importance was quantified by five-repeat permutation of each of the 500 genes across validation participants, with all other gene values unchanged. Predictions were averaged across three independently initialized genetic-branch models to reduce initialization-dependent variability. Gene importance was defined as the decrease from unperturbed ROC-AUC to the mean ROC-AUC across five permutations. Mean ROC-AUC decrease was used to rank genes; PR-AUC decreases and the number of folds with positive importance were retained as supporting measures.

To test whether cohort-specific gene-importance rankings converged at the biological-process level, the 500 model-input genes in each cohort were ranked by mean ROC-AUC decrease after permutation and analyzed separately using preranked *GSEA v4.4.0* [54] against the Molecular Signatures Database (*MSigDB*) [55] Gene Ontology Biological Process collection (*c5.go.bp.v2023.2.Hs.symbols.gmt*). Weighted enrichment with 1000 permutations, no identifier collapsing, and gene-set sizes of 15–500 was used. Pathway significance was assessed using the *GSEA* false-discovery-rate, with P_FDR_ < 0.05 considered significant. Because the ranking statistic reflected permutation importance rather than expression change, enrichment direction was not interpreted as transcriptional up- or down-regulation.

### 4.5. Regional MRI Occlusion and Integrative Region–Gene-Feature Analysis

Imaging importance was evaluated by separately zero-occluding each Desikan–Killiany region [56]. The atlas was aligned to the MRI reference geometry and nearest-neighbor resampled to a 48 × 56 × 48 grid, yielding 110 nonzero cortical and subcortical regions. Because MRI inputs were standardized within volume, zero corresponded to the standardized mean intensity. Regional importance was defined as the decrease in ROC-AUC after setting the region to zero relative to the original images. Mean ROC-AUC and PR-AUC decreases were averaged across folds; voxel counts were recorded, and the atlas served only as a perturbation mask.

For integrative analysis, each occluded image was passed through the imaging branch and IG-CVAE to generate occlusion-conditioned GFV128. Regional perturbation magnitude was additionally quantified as the mean Euclidean distance from the original GFV128 in the standardized IG-CVAE feature space. Within each fold, ridge regression (*α* = 1.0) was fitted using only training participants to map standardized observed GFV128 to the standardized expression of the 20 genes with the highest genetic-branch permutation importance. The fitted mapping was then applied to the original and occlusion-conditioned GFV128 of held-out validation participants. The primary region–gene-feature statistic was the mean absolute standardized shift (Equation (2)).

(2) $$S_{r,g}=\frac{1}{K}\sum_{k=1}^{K}\frac{1}{n_{k}}\sum_{i=1}^{n_{k}}|z\;\left({\hat{g}}_{i,g,k}^{\left(r\right)}\right)-z\;\left({\hat{g}}_{i,g,k}^{\left(0\right)}\right)|$$

Here, *K* = 5, *n_k_* is the validation-fold sample size, and ${\hat{g}}^{\left(r\right)}$ and ${\hat{g}}^{\left(0\right)}$ are the occluded and original projected values. This procedure yielded 2200 region–gene-feature effects per cohort (110 regions × 20 genes). These effects quantify how perturbation of a structural MRI region changes the model-projected feature of a disease-informative gene through the learned MRI → MFV128 → GFV128 relationship. They do not represent measured regional gene expression, direct gene regulation, or causal brain–gene relationships.

## 5. Conclusions

IG-CVAE showed that structural MRI can condition a blood-expression-derived gene-feature representation that retains AD/CN information across independently analyzed cohorts, although estimates from the small ANM paired test set should be interpreted cautiously. Diagnosis-matched participant-pair permutation analysis supported some MRI–GFV correspondence beyond diagnostic status, but participant-specific molecular correspondence remained limited. Molecular importance remained cohort-specific at the individual-gene levels, with no significant pathway-level convergence, whereas perturbation analyses showed stronger cross-cohort convergence in cerebral white matter and linked this shared anatomical signal within each cohort to distinct disease-informative molecular features. These findings support participant-paired imaging-transcriptomic modeling as a hypothesis-generating approach for investigating relationships between structural neurodegeneration and peripheral molecular variation in AD. The generated representations and region–gene-feature associations should not be interpreted as reconstruction of the measured transcriptome, regional gene expression, or causal MRI-to-gene relationships. In future work, artificial intelligence frameworks that integrate these imaging and molecular signals with direct measurements of gene regulation could help prioritize regulatory mechanisms and therapeutic hypotheses for experimental validation.

## Figures and Tables

**Table 1:** Participant characteristics by modality and cohort.

	ADNI					ANM				
	AD	CN	Total	*p* Value	Effect Sizes	AD	CN	Total	*p* Value	Effect Sizes
GE	N = 90	N = 212	N = 302			N = 181	N = 169	N = 350		
Age	76.58 ± 7.77	75.77 ± 6.64	76.01 ± 7.00	0.387	d = 0.116	77.17 ± 6.30	74.88 ± 5.80	76.06 ± 6.16	<0.001	d = 0.378
Sex: F	37.78% (34)	52.36% (111)	48.01% (145)	0.028	V = 0.133	69.06% (125)	61.54% (104)	65.43% (229)	0.172	V = 0.079
Sex: M	62.22% (56)	47.64% (101)	51.99% (157)			30.94% (56)	38.46% (65)	34.57% (121)		
APOE4: 0	33.33% (30)	73.58% (156)	61.59% (186)	2 × 10^−11^	V = 0.403	44.07% (78)	72.73% (120)	57.89% (198)	1 × 10^−7^	V = 0.304
APOE4: 1	48.89% (44)	23.58% (50)	31.13% (94)			42.37% (75)	24.24% (40)	33.63% (115)		
APOE4: 2	17.78% (16)	2.83% (6)	7.28% (22)			13.56% (24)	3.03% (5)	8.48% (29)		
Batch1	-	-	-	-		54.14% (98)	57.99% (98)	56.00% (196)	0.538	V = 0.039
Batch2	-	-	-	-		45.86% (83)	42.01% (71)	44.00% (154)		
Paired	N = 85	N = 204	N = 289			N = 46	N = 52	N = 98		
Age	76.33 ± 7.74	75.63 ± 6.60	75.84 ± 6.95	0.466	d = 0.101	75.22 ± 5.90	74.83 ± 5.62	75.01 ± 5.73	0.739	d = 0.068
Sex: F	38.82% (33)	51.96% (106)	48.10% (139)	0.056	V = 0.120	67.39% (31)	53.85% (28)	60.20% (59)	0.246	V = 0.138
Sex: M	61.18% (52)	48.04% (98)	51.90% (150)			32.61% (15)	46.15% (24)	39.80% (39)		
APOE4: 0	34.12% (29)	73.53% (150)	61.94% (179)	4 × 10^−10^	V = 0.385	34.78% (16)	71.15% (37)	54.08% (53)	0.001	V = 0.377
APOE4: 1	50.59% (43)	23.53% (48)	31.49% (91)			47.83% (22)	25.00% (13)	35.71% (35)		
APOE4: 2	15.29% (13)	2.94% (6)	6.57% (19)			17.39% (8)	3.85% (2)	10.20% (10)		
Batch1	-	-	-	-		93.48% (43)	96.15% (50)	94.90% (93)	0.888	V = 0.061
Batch2	-	-	-	-		6.52% (3)	3.85% (2)	5.10% (5)		
MRI	N = 631	N = 864	N = 1495			N = 56	N = 55	N = 111		
Age	74.69 ± 7.67	72.41 ± 7.11	73.37 ± 7.43	7 × 10^−9^	d = 0.309	75.16 ± 5.58	75.00 ± 5.52	75.08 ± 5.53	0.889	d = 0.029
Sex: F	42.79% (270)	55.79% (482)	50.30% (752)	9 × 10^−7^	V = 0.128	60.71% (34)	50.91% (28)	55.86% (62)	0.396	V = 0.099
Sex: M	57.21% (361)	44.21% (382)	49.70% (743)			39.29% (22)	49.09% (27)	44.14% (49)		
APOE4: 0	33.94% (206)	68.52% (531)	53.33% (737)	1 × 10^−43^	V = 0.377	37.50% (21)	70.91% (39)	54.05% (60)	0.001	V = 0.344
APOE4: 1	47.94% (291)	28.90% (224)	37.26% (515)			48.21% (27)	25.45% (14)	36.94% (41)		
APOE4: 2	18.12% (110)	2.58% (20)	9.41% (130)			14.29% (8)	3.64% (2)	9.01% (10)		

Values are mean ± standard deviation (SD) or N (%); *p* values compare AD and CN participants within each cohort. Effect sizes are reported as Cohen’s d for age and Cramér’s V for categorical variables. ADNI, Alzheimer’s Disease Neuroimaging Initiative; ANM, AddNeuroMed; AD, Alzheimer’s disease; CN, cognitively normal control; GE, gene expression; MRI, magnetic resonance imaging; APOE ε4, apolipoprotein E ε4 allele count; F, female; M, male. Paired denotes participants with both GE and MRI data. Batch1 and Batch2 denote ANM microarray batches.

**Table 2:** Cross-validation and independent test classification performance.

Model	Cohort	Cross-Validation		Independent Test	
		ROC-AUC	PR-AUC	ROC-AUC	PR-AUC
Genetic branch	ADNI	0.954 ± 0.046	0.905 ± 0.124	0.646 (0.500–0.785)	0.602 (0.481–0.783)
	ANM	0.888 ± 0.028	0.901 ± 0.029	0.851 (0.753–0.928)	0.865 (0.784–0.935)
Imaging branch	ADNI	0.911 ± 0.012	0.877 ± 0.026	0.942 (0.914–0.964)	0.922 (0.880–0.954)
	ANM	0.776 ± 0.064	0.812 ± 0.082	0.752 (0.529–0.934)	0.720 (0.544–0.942)
IG-CVAE	ADNI	0.938 ± 0.040	0.839 ± 0.117	0.939 (0.873–0.987)	0.933 (0.865–0.986)
	ANM	0.732 ± 0.077	0.774 ± 0.098	0.869 (0.677–0.980)	0.865 (0.704–0.980)

Cross-validation values represent held-out validation performance and are reported as mean ± standard deviation (SD) across five folds; independent test values are reported with 95% confidence intervals. ROC-AUC, area under the receiver operating characteristic curve; PR-AUC, area under the precision–recall curve; IG-CVAE, imaging-genetics conditional variational autoencoder; ADNI, Alzheimer’s Disease Neuroimaging Initiative; ANM, AddNeuroMed.

**Table 3:** Agreement between observed and MRI-generated GFV128.

Model	Cohort	Cross-Validation				Independent Test			
		MSE	MAE	Cosine Similarity	R^2^	MSE	MAE	Cosine Similarity	R^2^
IG-CVAE	ADNI	0.245 ± 0.033	0.330 ± 0.026	0.702 ± 0.053	0.387 ± 0.107	0.334	0.375	0.590	0.161
	ANM	0.280 ± 0.026	0.362 ± 0.015	0.650 ± 0.031	0.298 ± 0.047	0.321	0.393	0.589	0.211

Cross-validation values represent held-out validation performance and are reported as mean ± standard deviation (SD) across five folds; independent test values are point estimates. GFV128, 128-dimensional gene-feature vector; MRI, magnetic resonance imaging; MSE, mean squared error; MAE, mean absolute error; R^2^, coefficient of determination; IG-CVAE, imaging-genetics conditional variational autoencoder; ADNI, Alzheimer’s Disease Neuroimaging Initiative; ANM, AddNeuroMed.

## Data Availability

Data used in preparation of this article were obtained from the Alzheimer’s Disease Neuroimaging Initiative (ADNI) and AddNeuroMed (ANM) under their respective data-use agreements. The code is available at: https://github.com/xiaoqinghuanglab/IG-CVAE/ (accessed on 27 October 2026).

## Supplementary Materials

The following supporting information can be downloaded at: https://www.mdpi.com/article/10.3390/ijms27219741/s1.

## Abbreviations

The following abbreviations are used in this manuscript:

AD	Alzheimer’s disease
ADNI	Alzheimer’s Disease Neuroimaging Initiative
ANM	AddNeuroMed
APOE ε4	Apolipoprotein E ε4 allele
BIDS	Brain Imaging Data Structure
CN	Cognitively normal control
CVAE	Conditional variational autoencoder
FDR	False discovery rate
GE	Gene expression
GELU	Gaussian error linear unit
GFV128	128-dimensional gene-feature vector
GSEA	Gene-set enrichment analysis
IG-CVAE	Imaging-genetics conditional variational autoencoder
KL	Kullback–Leibler
MFV128	128-dimensional MRI-feature vector
MRI	Magnetic resonance imaging
MSE	Mean squared error
MAE	Mean absolute error
MSigDB	Molecular Signatures Database
PR-AUC	Area under the precision-recall curve
ROC-AUC	Area under the receiver operating characteristic curve
3D CNN	Three-dimensional convolutional neural network
