# Supplementary material for: An Explainable MRI-Conditioned Generative Framework for Gene-Feature Representations in Alzheimer’s Disease
Source: Int J Mol Sci. 2026 Oct 30;27(21):9741. doi: 10.3390/ijms27219741 (PMC13763474; doi:10.3390/ijms27219741)
Supplement: Supplementary file 1 [file ijms-27-09741-s001.zip › Supplementary Figure S1.pdf]

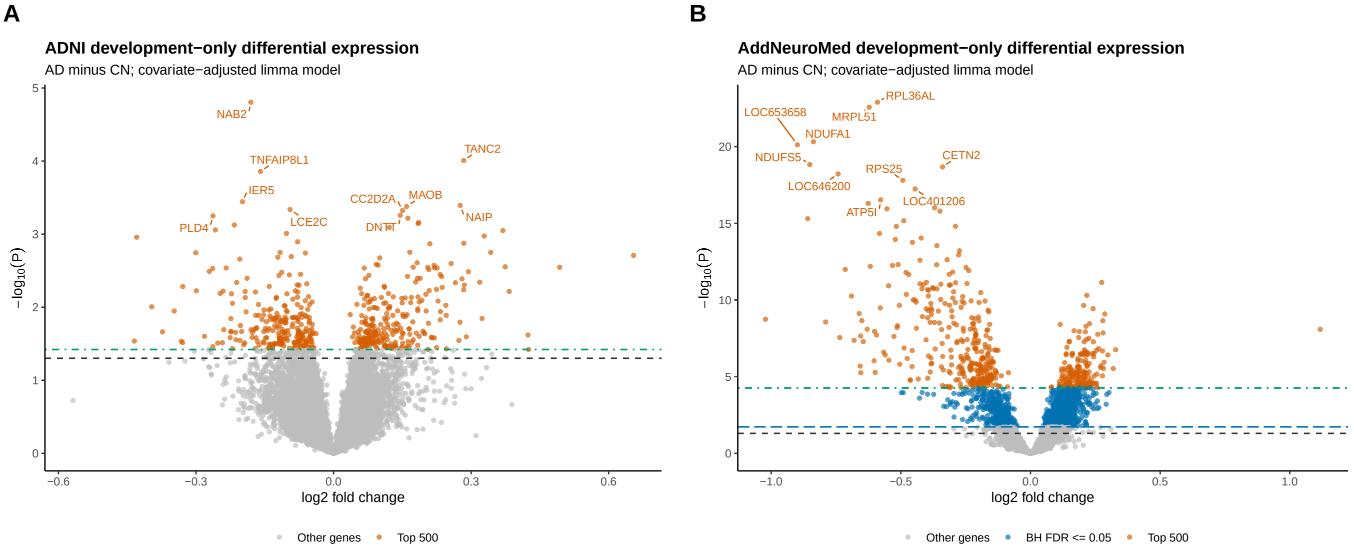

A
ADNI development−only differential expression
AD minus CN; covariate−adjusted limma model
−log10(P)
log2 fold change
−0.6
−0.3
0.0
0.3
0.6
0
1
2
3
4
5
NAB2
TNFAIP8L1
IER5
PLD4
LCE2C
CC2D2A
MAOB
DNTT
TANC2
NAIP
Other genes
Top 500
B
AddNeuroMed development−only differential expression
AD minus CN; covariate−adjusted limma model
−log10(P)
log2 fold change
−1.0
−0.5
0.0
0.5
1.0
0
5
10
15
20
RPL36AL
LOC653658
MRPL51
NDUFA1
NDUFS5
LOC646200
CETN2
RPS25
LOC401206
ATP5I
Other genes
BH FDR <= 0.05
Top 500
