# Supplementary material for: An Explainable MRI-Conditioned Generative Framework for Gene-Feature Representations in Alzheimer’s Disease
Source: Int J Mol Sci. 2026 Oct 30;27(21):9741. doi: 10.3390/ijms27219741 (PMC13763474; doi:10.3390/ijms27219741)
Supplement: Supplementary file 1 [file ijms-27-09741-s001.zip › Supplementary GenAI.pdf]

AI tool: OpenAI ChatGPT
Model version(s): GPT-5.6 Sol Light (Chat), GPT-6 (Chat)

1. Purpose of AI Assistance

ChatGPT was used to assist with manuscript language refinement, identifying coding errors and suggesting coding corrections. The authors reviewed and revised the AI-assisted outputs and retained responsibility for the final manuscript, code, and scientific findings.

2. Representative Prompts

The following are representative prompts used during manuscript preparation and code development.

A. Manuscript preparation

Prompt: "we next asked whether structural MRI perturbation revealed a more reproducible cross-cohort anatomical substrate and whether that substrate was linked to disease-informative molecular features.

make it short."

Prompt: "Cohort-specific selection was used because ADNI and ANM were profiled using different expression platforms and did not provide identical transcriptomic feature spaces; a common gene panel was therefore not imposed across cohorts.

this sentence itself repeats the same info, make it concise"

Prompt: "Cohort-specific selection was used because ADNI and ANM used different microarray platforms, workflows, and non-identical transcriptomic feature spaces.

what if I say this"

Prompt: "The two top-500 panels shared 13 genes, of which 12 (92.3%) showed the same direction of differential expression in AD versus CN across both cohorts. Thus, although gene prioritization was largely cohort-specific, the small subset selected in both datasets showed strong directional concordance.

What if blend these 2 sentences"

Prompt: "To determine whether these cohort-specific gene-importance profiles converged write like thisAt the pathway level, no Gene Ontology Biological Process term met the FDR $q < 0.05$ significance threshold in either ADNI or ANM (Table S8). Thus, the cohort-specific molecular importance observed at the gene level did not resolve into a reproducible pathway-level signature.

I will keep this if this is grammatically correct"

Prompt: "This model-specific cohort design allowed the Gene and MRI branches to use all participants with the required modality, while participants with both gene-expression and MRI data formed the paired cohort used to train and evaluate XIG-CVAE.

I think this is good but need slight changes: with the required modality this part sounds vauge”

B. Coding assistance for error identification

Prompt: “(xigcvae_gpu) [mshihab@g1 Project_folder]$ python -m py_compile scripts/06_run_cvae.py
(xigcvae_gpu) [mshihab@g1 Project_folder]$ python scripts/06_run_cvae.py VALIDATE --cohort ALL
Traceback (most recent call last):
File "/N/u/mshihab/Quartz/venvs/xigcvae_gpu/lib/python3.12/site-packages/pandas/core/indexes/base.py", line 3641, in get_loc
return self._engine.get_loc(casted_key)
^^^^^^^^^^^^^^^^^^^^^^^^^^^^^^^^
File "pandas/_libs/index.pyx", line 168, in pandas._libs.index.IndexEngine.get_loc
File "pandas/_libs/index.pyx", line 197, in pandas._libs.index.IndexEngine.get_loc
KeyError: 'cv_fold_mri_branch'
The above exception was the direct cause of the following exception:
Traceback (most recent call last):
File "/N/project/SingleCell_Image/Ronak/Project_folder/scripts/06_run_cvae.py", line 435, in <module>
main()
File "/N/project/SingleCell_Image/Ronak/Project_folder/scripts/06_run_cvae.py", line 430, in main

What is this error”

Prompt: “[mshihab@g1 Project_folder]$ ./.venv/bin/python scripts/04_run_gene_branch.py --help
anch.py VALIDATE --cohort ALL
^C
Traceback (most recent call last):
File "/N/project/SingleCell_Image/Ronak/Project_folder/scripts/04_run_gene_branch.py", line 24, in <module>
import pandas as pd
File "/N/project/SingleCell_Image/Ronak/Project_folder/.venv/lib/python3.12/site-packages/pandas/__init__.py", line 46, in <module>
from pandas.core.api import (
File "/N/project/SingleCell_Image/Ronak/Project_folder/.venv/lib/python3.12/site-packages/pandas/core/api.py", line 27, in <module>
from pandas.core.arrays import Categorical
File "/N/project/SingleCell_Image/Ronak/Project_folder/.venv/lib/python3.12/site-packages/pandas/core/arrays/__init__.py", line 1, in <module>
from pandas.core.arrays.arrow import ArrowExtensionArray

it get stuck”

Prompt: “(xigcvae_gpu) [mshihab@g1 Project_folder]$

(xigcvae_gpu) [mshihab@g1 Project_folder]$ python -m py_compile scripts/05_run_mri_branch.py
scripts/05_run_mri_branch.py VALIDATE --cohort ALL(xigcvae_gpu) [mshihab@g1 Project_folder]$ python scripts/05_run_mri_branch.py VALIDATE --cohort ALL
Traceback (most recent call last):
File "/N/project/SingleCell_Image/Ronak/Project_folder/scripts/05_run_mri_branch.py", line 166, in <module>
main()
File "/N/project/SingleCell_Image/Ronak/Project_folder/scripts/05_run_mri_branch.py", line 152, in main
ap=argparse.ArgumentParser(description="Leakage-controlled dual-cohort MRI branch.")
^^^^^^^^
NameError: name 'argparse' is not defined. Did you forget to import 'argparse'?
(xigcvae_gpu) [mshihab@g1 Project_folder]$

What is the issue here”

Prompt: “Traceback (most recent call last):
File "<stdin>", line 7, in <module>
File "<frozen importlib._bootstrap_external>", line 995, in exec_module
File "<frozen importlib._bootstrap>", line 488, in _call_with_frames_removed
File "/N/project/SingleCell_Image/Ronak/Project_folder/scripts/05_run_mri_branch.py", line 27, in <module>
@dataclass(frozen=True)
^^^^^^^^^^^^^^^^^^^^^^
File "/N/soft/rhel8/python/gnu/3.12.4/lib/python3.12/dataclasses.py", line 1265, in wrap
return _process_class(cls, init, repr, eq, order, unsafe_hash,
^^
File "/N/soft/rhel8/python/gnu/3.12.4/lib/python3.12/dataclasses.py", line 983, in wrap
and _is_type(type, cls, dataclasses, dataclasses.KW_ONLY,
^^^
File "/N/soft/rhel8/python/gnu/3.12.4/lib/python3.12/dataclasses.py", line 749, in _is_type
ns = sys.modules.get(cls.__module__).__dict__
^^
AttributeError: 'NoneType' object has no attribute '__dict__'. Did you mean: '__dir__'?
(xigcvae_gpu) [mshihab@g1 Project_folder]$

why is the dataclass import failing”
