# Supplementary material for: An Explainable MRI-Conditioned Generative Framework for Gene-Feature Representations in Alzheimer’s Disease
Source: Int J Mol Sci. 2026 Oct 30;27(21):9741. doi: 10.3390/ijms27219741 (PMC13763474; doi:10.3390/ijms27219741)
Supplement: Supplementary file 1 [file ijms-27-09741-s001.zip › Supplementary Method.pdf]

Supplementary Method S1. Diagnosis-matched participant-pair permutation analysis

To assess whether MRI-generated GFV128 showed participant-specific correspondence beyond shared AD/CN status, a diagnosis-matched participant-pair permutation analysis was performed using only the paired participants in the five-fold cross-validation set. Within each cross-validation training fold, MRI-derived MFV128 order was held fixed while GFV128 training targets were reassigned to a different participant within the same diagnosis group, thereby preserving AD/CN composition while disrupting the correct participant-level MRI–GFV pairing. IG-CVAE was retrained for each permutation using the same fold assignments, branch representations, scaling procedure, architecture, loss, hyperparameters, and initialization as in the primary analysis, while validation participants retained their correct MRI-GFV pairings. One hundred diagnosis-matched permutations were performed. Generated-observed agreement was quantified using MSE, MAE, cosine similarity, and R^2, and retained AD/CN information was assessed using ROC-AUC and PR-AUC. The correctly paired model metrics were compared with the corresponding permutation distributions using one-sided empirical P values. Independent test participants were not accessed in this analysis.

Supplementary Method S2. Covariate-adjusted IG-CVAE sensitivity analysis

To assess the potential influence of demographic and genetic covariates on the learned MRI-GFV relationship, a sensitivity analysis was performed adjusting for age, sex, and APOE ε4 allele count. Within each of the five cross-validation folds, linear covariate models were fitted separately to MFV128 and GFV128 using training participants only and were used to remove the estimated covariate effects before IG-CVAE training and evaluation. The fitted transformations were applied unchanged to the corresponding held-out validation participants. Adjusted MFV128 and GFV128 representations were subsequently scaled using parameters estimated from the training participants only. IG-CVAE then used the same participants, folds, frozen branch representations, architecture, optimization procedure, and evaluation metrics as the primary analysis. After cross-validation, final covariate-adjustment models and feature scalers were fitted using the complete train-validation set, and the final covariate-adjusted IG-CVAE was trained using the same final-training procedure as the primary analysis. For independent-test evaluation, the frozen covariate-adjustment models, scalers, IG-CVAE, and diagnostic classifier were applied unchanged to the locked independent test participants; no covariate fitting, model refitting, or tuning was performed using the independent test set.

Supplementary Method S3. Deterministic MRI-to-GFV mapping benchmark

A deterministic multilayer perceptron (MLP) was implemented as a direct cross-modal mapping baseline using the same fold-specific MRI-derived MFV128 inputs and observed GFV128 targets used for IG-CVAE. The network mapped MFV128 through two 256-unit hidden layers to a 128-dimensional GFV output; each hidden layer used layer normalization, GELU activation, and dropout (0.10). Within each of the five cross-validation folds, MFV128 and GFV128 were standardized separately using *StandardScaler* objects fitted only on the training participants and applied unchanged to the corresponding held-out validation participants. The MLP was optimized using mean squared error loss and AdamW (learning rate, 1×10^{-3}; weight decay, 1×10^{-4}; batch size, 32) for up to 200 epochs, with early stopping after 20 epochs without improvement in validation MSE and gradient clipping at norm 5.0. Five-fold cross-validation used the same fixed folds and fold-matched frozen branch representations as IG-CVAE. After cross-validation, final MFV128 and GFV128 scalers were fitted using the complete train-validation set, and a final MLP was trained on the complete train-validation set for the mean best epoch identified across the five folds. The resulting scalers and MLP were then applied unchanged to the locked independent test set, with no test-set fitting or tuning. Generated-observed GFV128 agreement was evaluated using MSE, MAE, cosine similarity, and R^2, matching the IG-CVAE agreement metrics.
